# Systemic Lupus Erythematosus: How Machine Learning Can Help Distinguish between Infections and Flares

**DOI:** 10.3390/bioengineering11010090

**Published:** 2024-01-17

**Authors:** Iciar Usategui, Yoel Arroyo, Ana María Torres, Julia Barbado, Jorge Mateo

**Affiliations:** 1 Department of Internal Medicine, Hospital Clínico Universitario, 47005 Valladolid, Spain; iusateguim@gmail.com; 2 Department of Technologies and Information Systems, Faculty of Social Sciences and Information Technologies, Universidad de Castilla-La Mancha (UCLM), 45600 Talavera de la Reina, Spain; yoel.arroyo@uclm.es; 3 Medical Analysis Expert Group, Institute of Technology, Universidad de Castilla-La Mancha (UCLM), 16071 Cuenca, Spain; ana.torres@uclm.es; 4 Medical Analysis Expert Group, Instituto de Investigación Sanitaria de Castilla-La Mancha (IDISCAM), 45071 Toledo, Spain; 5 Department of Internal Medicine, Hospital Universitario Río Hortega, 47012 Valladolid, Spain; juliabarbado@yahoo.es

**Keywords:** Systemic Lupus Erythematosus, medical treatment, machine learning, artificial intelligence

## Abstract

Systemic Lupus Erythematosus (SLE) is a multifaceted autoimmune ailment that impacts multiple bodily systems and manifests with varied clinical manifestations. Early detection is considered the most effective way to save patients’ lives, but detecting severe SLE activity in its early stages is proving to be a formidable challenge. Consequently, this work advocates the use of Machine Learning (ML) algorithms for the diagnosis of SLE flares in the context of infections. In the pursuit of this research, the Random Forest (RF) method has been employed due to its performance attributes. With RF, our objective is to uncover patterns within the patient data. Multiple ML techniques have been scrutinized within this investigation. The proposed system exhibited around a 7.49% enhancement in accuracy when compared to k-Nearest Neighbors (KNN) algorithm. In contrast, the Support Vector Machine (SVM), Binary Linear Discriminant Analysis (BLDA), Decision Trees (DT) and Linear Regression (LR) methods demonstrated inferior performance, with respective values around 81%, 78%, 84% and 69%. It is noteworthy that the proposed method displayed a superior area under the curve (AUC) and balanced accuracy (both around 94%) in comparison to other ML approaches. These outcomes underscore the feasibility of crafting an automated diagnostic support method for SLE patients grounded in ML systems.

## 1. Introduction

Systemic Lupus Erythematosus (SLE) is a chronic autoimmune affliction that affects various physiological systems. It serves as an exemplary autoimmune disorder, and its intricate nature poses significant challenges. The varied clinical presentations of SLE, coupled with distinct complexities in both diagnosis and treatment, present a formidable task for healthcare professionals. The emergence of multiple mechanisms results in the breakdown of self-tolerance and subsequent organ dysfunction. Progress in elucidating the molecular and cellular foundations of this condition, in conjunction with the identification of genetic variations, contributes to a more profound comprehension of its pathogenesis, offering promise for therapeutic advancements in the near future.

Commonly known as lupus, it varies in prevalence depending on geographic location, ethnicity, and research study design. In the United States, an estimated 241 cases per 100,000 adults have been reported, while in Spain, the updated figure is 210 cases per 100,000 inhabitants [1]. The Lupus Foundation of America estimates that approximately 161,000 to 322,000 individuals in the U.S. are affected by SLE, translating to a prevalence of approximately 0.05% to 0.1% of the population. Predominantly, it affects young, fertile females and has resulted in increased mortality, although improved treatment modalities have positively impacted survival rates. Notably, the onset of the disease frequently occurs during the childbearing years. Certain demographic groups, including women, people of color (particularly African American, Hispanic, and Asian populations), and individuals of reproductive age, may experience higher prevalence rates. Simultaneously, several factors contribute to a state of relative immunodeficiency in individuals with SLE, including aging, the increasing use of targeted biologic therapies, and the chronic nature of the disease. Furthermore, the presence of other comorbidities such as malignancy, infections, malnutrition, and more further compounds the complexity of the disease. SLE is a complex and heterogeneous condition, manifesting symptoms across a spectrum from mild to severe. The precise etiology of SLE remains not fully understood, with its development believed to result from a combination of genetic and environmental factors. Moreover, the prevalence of SLE may undergo changes over time, influenced by factors such as improvements in diagnostic methods and increased awareness of the disease. Collectively, these multifaceted factors underscore the need for a comprehensive understanding of the diverse epidemiological and clinical aspects of SLE to inform effective management strategies and interventions.

Emerging evidence suggests that immunodeficiency and systemic autoimmunity are interconnected manifestations of a shared underlying process [2]. Immune disorders present as both susceptibility to infections and autoimmune symptoms, indicating a dual impact on the immune system—reduced ability to clear infections and a disruption of self-tolerance. On the other hand, infections are one of the most common causes of death and are often associated with high levels of activity in SLE. Early diagnosis of immunodeficency in SLE is the first step to contribute to detect infections, which are likely to be associated with flares, allows prompt initiation of treatment, a better prognosis, and a reduction in organ dysfunction [3,4,5,6,7]. In the absence of specific criteria that can differentiate between a severe infection and an exacerbation in SLE, the development of clinical studies and guidelines becomes imperative to facilitate a more precise classification of these patients [8].

In pursuit of this objective, Machine Learning (ML) draws inspiration from biological nervous systems. Its fundamental principle revolves around presenting algorithms with input data, subjecting them to computer analysis to predict output values within an acceptable range of accuracy, recognizing data patterns and trends, and ultimately assimilating knowledge from prior experiences [9]. ML delves into intricate data distributions, establishes probabilistic relationships, and identifies the minimum set of features required to capture essential data patterns through repeated cross-validation, culminating in the formulation of predictive models. Numerous studies have leveraged ML methods to develop more precise diagnostic algorithms for stratifying autoimmune diseases, thereby preventing or mitigating observed morbidity [10]. ML methods consistently exhibit superior performance compared to traditional statistical models [9,11,12,13]. A variety of ML techniques, including Support Vector Machine (SVM), Binary Linear Discriminant Analysis (BLDA), k-Nearest Neighbors (KNN), and Decision Trees (DT) [14,15,16,17], have been employed for data analysis. These systems represent a selection of algorithms designed for classifying data and processing information, and they have been explored in the context of various autoimmune diseases, including SLE, rheumatoid arthritis, lupus tubulointerstitial inflammation, and neuropsychiatric SLE [18,19,20,21,22,23].

In this paper, we present a system that utilizes the Random Forest (RF) method for the analysis of immunodeficiency patterns in SLE patients. RF is an ML algorithm that operates by constructing a multitude of decision trees for classification and prediction. For its capacity to enhance accuracy and processing speed, and several notable advantages, including a low computational burden, flexibility in model tuning, high scalability, and algorithmic optimization, it serves as the cornerstone of this approach. Through the application of RF, we aim to predict the immunodeficiency status of our patients, with the overarching goal of not only identifying optimal treatment options but also designing personalized preventive measures and tailoring patient-specific follow-up strategies.

The paper is structured as follows. The first section outlines the topic, purpose, and significance of this study. Second section introduces a detailed description of material and methods. Third section entails the main findings of the study, including data, analysis, and interpretation of the results obtained. Fourth section explores a discussion of these results. And finally, the paper concludes with a summary of the research and some concluding remarks.

## 2. Materials and Methods

### 2.1. Materials

The study cohort included 125 patients who met the American College of Rheumatology criteria for SLE in 2019 [23]. These individuals were enrolled from the Autoimmune Unit Registry at Valladolid Clinic Hospital (HCUV) between 2017 and 2019. The experimental protocol adhered to the principles outlined in the Declaration of Helsinki (2008) and received approval from the Clinical Research Ethics Committee of the HCUV. The study was conducted in compliance with Spanish data protection laws (LO 15/1999) and specifications (RD 1720/2007).

Consequently, a retrospective review of patients was systematically conducted, encompassing the collection of epidemiological, analytical, immunological, and clinical characteristics. Relevant immunological parameters for evaluating immune competence included leucocytes, neutrophils, CD3, CD4 and CD8 T-cell counts, CD19 B-cell and Natural Killer (NK) cell levels, serum immunoglobin isotypes (IgG, IgA, IgM), IgG subclasses, and complement levels (C3, C4). Exclusion criteria involved patients with evidence of active disease (SLEDAI >= 4) or significant residual proteinuria (>500 mg). Following this selection strategy, 31 patients were excluded from the study.

Flow cytometry was performed to identify cell populations. Serum levels of immunoglobulin isotypes and IgG subclasses and complement were determined by nephelometry. Standardized reference ranges from the immunology laboratory of our institution were used to define control patients. Laboratory levels below the reference ranges were considered as possible immunodeficiency status: leucocytes < 4000 cL/μL, neutrophils < 1800 cL/μL, lymphocytes < 1500 cL/μL, CD3 T-cell < 700 cL/μL, CD19 B-cell < 100 cL/μL, CD4 T-cell < 300 cL/μL, CD8 T-cell < 200 cL/μL, NK cell < 90 cL/μL, IgG < 870 mg/dL, IgG1 < 383 mg/dL, IgG2 < 242 mg/dL, IgG3 < 22 mg/dL, IgG4 < 4 mg/dL, IgA < 117 mg/dL, IgM < 60 mg/dL, C3 < 90 mg/dL, C4 < 10 mg/dL; special data for patients between 14 and 18 years old were: IgG1 < 315 mg/dL, IgG2 < 242 mg/dL, IgG3 < 23 mg/dL, IgG4 < 11 mg/dL. Severe infection was defined as infection which required hospitalization of seriousness, treatment needed or recommended monitoring.

### 2.2. Method

This study introduces an ML method centered on the Random Forest (RF) algorithm. RF, a widely adopted ML algorithm within supervised learning, is applied for both classification and regression challenges in ML. Renowned for its simplicity, versatility, and robustness, RF embodies a potent ML algorithm with several noteworthy attributes: (1) operative as an ensemble learning approach, it combines decisions from multiple models to improve overall performance; (2) employing decision trees as base-level models; (3) mitigating overfitting by averaging results across several trees, thereby diminishing the risk of developing complex models performing well on training data but poorly on new data; (4) adeptly handling missing values by learning the optimal imputation value based on the reduction in the utilized criterion; (5) furnishing a reliable estimate of the importance of variables in the classification process; (6) demonstrating flexibility in its applicability to both regression and classification tasks; and (7) executing swiftly with minimal preprocessing requirements compared to alternative algorithms, capable of handling categorical variables without necessitating the creation of dummy variables. Consequently, RF is the chosen algorithm for crafting the model aimed at detecting immunodeficiency patterns within the SLE population [24,25].

Given a dataset $S=\{x_{j},y_{j}\}$ , where $x_{j}$ represents feature vectors and $y_{j}$ corresponds to labels, the RF algorithm proceeds as follows:

For each of the *n* trees in the forest:

1. Draw a bootstrap sample $Z^{*}$ of size *N* from the training data.
2. Grow a decision tree $T_{b}$ to the bootstrapped data by recursively repeating the following steps for each terminal node of the tree, until the minimum node size $n_{\min}$ is reached:
  (a) Select *m* variables at random from the *p* variables.
  (b) Pick the best variable/split-point among the *m* variables.
  (c) Split the node into two daughter nodes.

The prediction of the RF then aggregates the predictions of the *n* trees.

For regression, it is typically the average over all trees:

(1) $${\hat{f}}_{rf}\left(x\right)=\frac{1}{n}\sum_{b=1}^{n}T_{b}\left(x\right)$$

For classification, it is determined by the majority vote:

(2) $${\hat{C}}_{rf}\left(x\right)=\mathrm{majority}{\left\{{\hat{C}}_{b}\left(x\right)\right\}}_{1}^{n}$$

Here, $T_{b}\left(x\right)$ and ${\hat{C}}_{b}\left(x\right)$ represent the prediction of the *b*-th decision tree for regression and classification, respectively.

The algorithm was designed and developed using Matlab software (MatLab 2023a, The Mathworks Inc., Natick, MA, USA). Furthermore, the proposed system underwent analysis alongside other ML systems prevalent in the scientific community. These included Support Vector Machine (SVM) [14], Binary Linear Discriminant Analysis (BLDA) [26], Decision Trees (DT) [15], Linear Regression (LR) [27,28], and k-Nearest Neighbor (KNN) [16] to assess its performance. Within the ML system’s learning process, it is imperative to control overtraining. To address this, the k-fold cross-validation technique was employed in our case.

**Figure 1:**
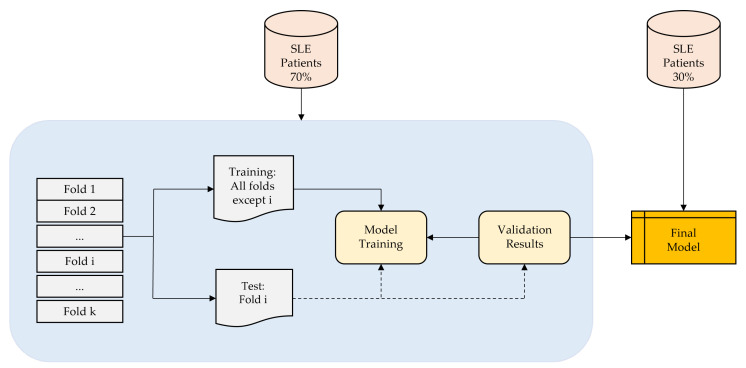
The figure shows the processes followed in this study for the classification of patients with SLE.

As depicted in Figure 1, each iteration involves the random classification of 70% of the patients for training and 30% for testing and validation. Notably, patient data are not shared between the training and validation subsets to prevent the algorithm from being validated with data from the same patients used in the training phase.

Additionally, techniques for hyperparameter optimization have been applied to fine-tune the hyperparameters of the methods. These hyperparameter values are adjusted during the training phase to maximize the accuracy of the ML method. The hyperparameters subjected to optimization encompass variables such as apprentices, neighbors, distance metric, distance weight, kernel, box constraint level, and multiclass method, each tailored to the specific requirements of the method in use. Bayesian optimization was chosen as the technique to enhance the performance of the various methods by optimizing the selection of diverse hyperparameters. Recall value and AUC were utilized as performance metrics. The entire study was iterated 100 times to obtain mean values and standard deviations for the process. Importantly, it should be emphasized that data used in each iteration were randomized, mitigating noise in the samples and ensuring the acquisition of results with statistically valid values [29].

### 2.3. Performance Evaluation

For this study, the most well-known metrics in artificial intelligence were implemented to test the performance of the methods [29]: balanced accuracy (BA), recall, precision, specificity (SP), degenerated Younden’s index (DYI) [29], receiver operating characteristic (ROC) and area under the curve (AUC). The F_1_ score is established as:

(3) $$F_{1}score=2\frac{Precision\cdot Recall}{Precision+Recall}$$

To test the classification performance of the model, the Matthew correlation coefficient (MCC) has been used, which is described as follows:

(4) $$MCC=\frac{TP\cdot TN-FP\cdot FN}{\sqrt{\left(TP+FP)(TP+FN)(TN+FP)(TN+FN\right)}}$$

where TP is the number of true positives, FP the number of false positives, TN the number of true negatives and FN the number of false negatives. And finally, Cohen’s Kappa (CK), CK is another metric that estimates the performance of the model [29].

## 3. Results

The study was conducted on a group of 125 patients diagnosed with SLE. Out of these, 94 patients met the specific criteria of having a SLEDAI-2K score of less than four points, and were thus included in the study. Further analysis revealed that 77 of these 94 patients showed signs of immunodeficiency. This means that approximately 81.9% of the patients with a SLEDAI-2K score less than four exhibited signs of immunodeficiency.

The cohort of patients had a median age of 52 years, whilst the median age at diagnosis was 38 years. The group was predominantly female, with 68 female patients compared to 9 male patients. The median duration of the disease among these patients was 14 years. At the time of data collection (see Table 1), 50 patients (64.9%) were being treated with corticosteroids at an average daily dose of 2.57 mg. In addition, 25 patients (34.9%) were receiving immunosuppressants such as azathioprine, methotrexate, and mycophenolate. Two patients were on belimumab treatment. Notably, none of the patients were undergoing treatment with rituximab.

In turn, 41 patients (53.2%) exhibited patterns of immunodeficiency. Among these patients, there were a total of 51 episodes of severe infections. The breakdown of these infections is as follows:

- 17 patients were hospitalized due to lower respiratory infections.
- 4 patients were hospitalized for upper respiratory infections.
- 9 patients were treated for urinary infections.
- 10 patients had soft tissue infections.
- 4 patients suffered from digestive infections.
- 1 patient was diagnosed with tuberculous lymphadenitis.

Table 1 provides an overview of the characteristics of patients exhibiting immunodeficiency patterns. The patients under study demonstrated a decline in the count of several immune cells. This was particularly evident in the case of NK cells, a component of the innate immune system, and CD19 B-cells, a part of the adaptive immune system. The latter includes IgG subclasses and IgM, both of which also showed a decrease. These patients exhibited reduced levels of various immune cells, as illustrated in Table 1, with notable decreases observed in NK cells within the innate immune system and CD19 B-cells within the adaptive immune system, including IgG subclasses and IgM.

The study employed a range of ML techniques to discern patterns of innate and adaptive immunodeficiency within the SLE population. The findings derived from these techniques, coupled with several ML algorithms for identifying immunodeficiency, are detailed below. Performance metrics such as BA, recall, specificity, precision, and AUC for the investigated ML methods are exhibited in Table 2 and Table 3. Both tables provide a detailed summary of performance metrics for different ML methods applied to variables IgG, IgG2, IgG3, IgG4 (Table 2), and IgM, NK, CD19, CD3 (Table 3). These variables are associated with immunoglobulins and immune cell populations, whilst the ML methods evaluated include SVM, BLDA, DT, KNN, and the RF proposed method. The results offer insights into how well each ML method performs in predicting or classifying the specified immunological variables, providing a comparative analysis of their strengths in terms of these metrics. The comprehensive nature of the data facilitates an informed selection of the most suitable method for each variable based on the desired performance criteria. Of particular note is the RF proposed method, which consistently outperforms across all variables, achieving the highest accuracy. KNN also demonstrates strong performance, particularly in IgM and CD3. LR were the lowest results obtained, whilst SVM, BLDA, and DT generally exhibit competitive results but with slightly lower accuracy than RF and KNN. In summary, the evaluation underscores the robust performance of the proposed method across the variables related to immunoglobulins and immune cell types, being the preferred model for classifying SLE patients due to its consistently high accuracy, balanced performance metrics, ensemble learning strengths, and robustness to noisy data observed.

Moreover, Table 4 and Table 5 present performance metrics, including F_1_ score, MCC, DYI, and Kappa values, for the ML methods applied. The observed values provide insights into the models’ effectiveness in classifying SLE patients. Thus, in Table 4 (variables IgG, IgG2, IgG3, and IgG4), RF consistently outperforms again other methods across all metrics, exhibiting high F_1_ score, MCC, DYI, and Kappa values. This suggests RF’s robustness in achieving a balanced trade-off between precision and recall, capturing the model’s ability to handle both positive and negative instances effectively. Again, KNN also shows competitive performance, while SVM, BLDA, and DT demonstrate slightly lower performance across these metrics, being LR the one which obtained the lowest performance values. Similar trends are observed in the variables related to immune cell types in Table 5 (IgM, NK, CD19, and CD3), where RF again demonstrates superior performance, especially notable in achieving high F_1_ score and DYI values. This reinforces RF’s suitability for SLE classification, indicating its ability to maintain a balance between true positives, true negatives, false positives, and false negatives. KNN also perform well, but RF consistently stands out as the top-performing model across the diverse set of variables.

**Figure 2:**
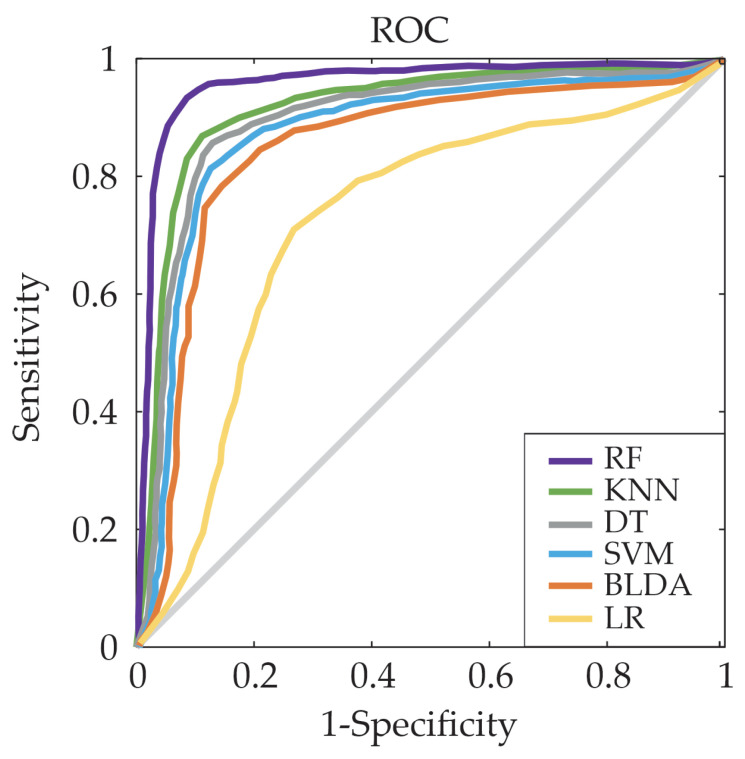
Example of ROC curve for the five assessed ML predictors for variable CD19.

For a comprehensive view of the trade-off between the true/false positive rates between the proposed system and other ML methods, the Receiver Operating Characteristic (ROC) curves were also generated. With this purpose in mind, the ROC curve is employed to quantify sensitivity and 1-specificity at various threshold levels. As illustrated in Figure 2, which shows the ROC curve for CD19 variable as example, the system that utilizes RF generates the largest area under the curve, indicating a superior level of predictive accuracy.

**Figure 3:**
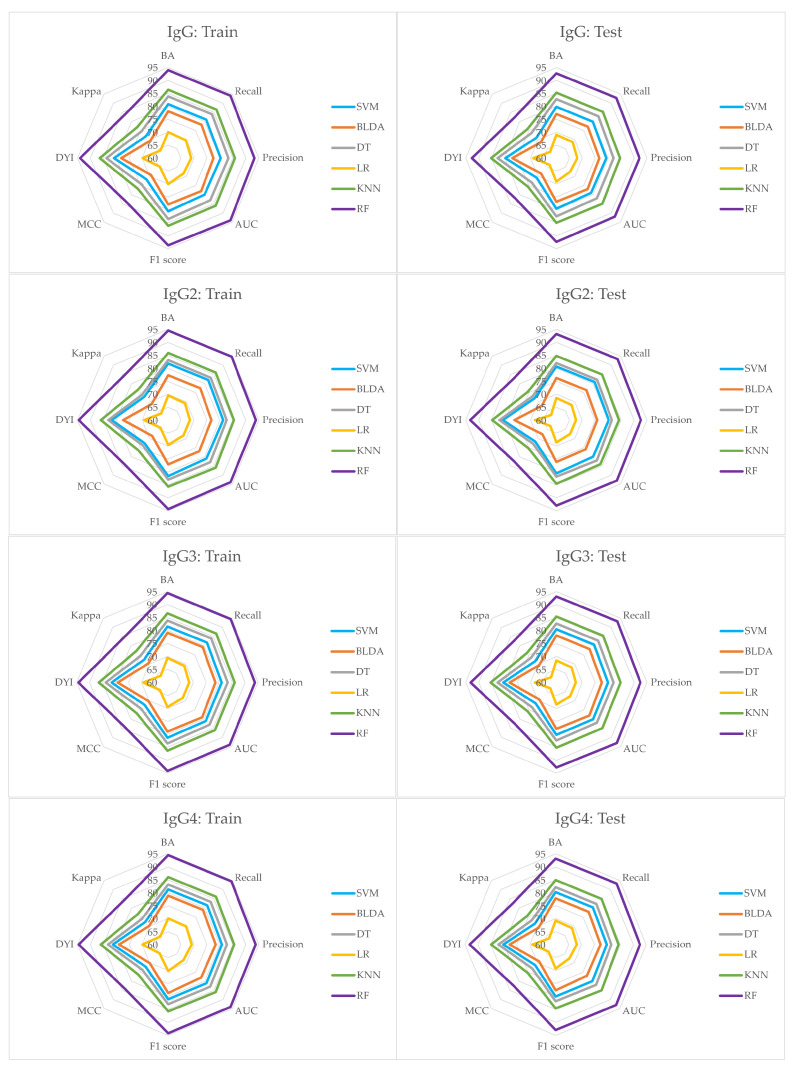
The figure shows the radar plots of the variables IgG, IgG2, IgG3 and IgG4, respectively.

**Figure 4:**
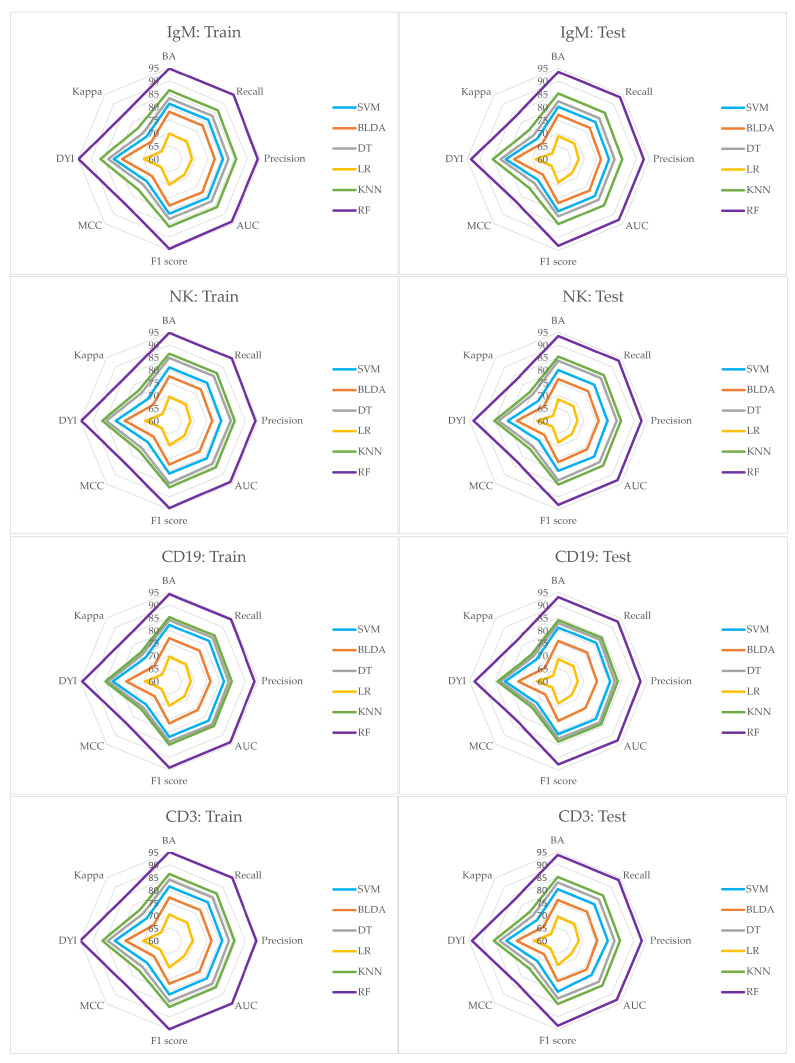
The figure shows the radar plots of the variables IgM, NK, CD19 and CD3, respectively.

In the study conducted, it was also observed that the subsets used for training the model exhibited high scores in the training metrics. When these models were tested, they showed a noticeable decrease in their scores. Nonetheless, as depicted in Figure 3 and Figure 4, the RF system emerges as a well-calibrated model, attaining an optimal point in training without succumbing to overfitting or underfitting. This approach consistently delivers accurate predictions for novel inputs. The RF system’s superior performance is evident, where it surpasses other methods by covering a larger area in the radar plots in both the training and testing phases.

## 4. Discussion

The task of managing patients with SLE is crucial in order to reduce the risk of irreversible organ damage [30,31]. This is not only vital for maintaining the health-related quality of life of the patients [32,33], but also for managing the direct costs associated with the treatment of SLE [34,35]. However, this task presents significant challenges due to the heterogeneous nature of SLE, which is characterized by variations in disease progression [36,37]. There is therefore an urgent need to improve the accuracy and classification of SLE flares, taking into account that the trigger of activity may be an infection in a situation of immunodeficiency. Numerous studies have been conducted to address this need, including recent research that has emerged over the last few years [31,33]. These studies have emphasized potential treatments for severe lupus manifestations such as lupus nephritis [31]. Despite the existence of several therapeutic agents in SLE, the disease continues to cause significant morbidity [31]. It is encouraging that a variety of therapeutic options are currently under investigation [31].

In clinical practice, the manifestation of a malar rash, coupled with the detection of anti-DNA autoantibodies in patients, often guides healthcare professionals towards the diagnosis of SLE [38,39]. It is noteworthy that SLE is characterized by a significant degree of phenotypic diversity, which includes both systemic and localized forms. The evolution of immunological and clinical features over time underscores the dynamic nature of this disease [33,40].

A multitude of models have been established to estimate the probability of SLE occurrence, providing a degree of confidence in differentiating it from other rheumatological disorders. These models leverage unsupervised clustering based on the nature and abundance of features, mirroring diagnostic reasoning, especially during initial patient consultations [41,42]. Certain models incorporate gene analysis techniques to improve the classification of SLE patients [19]. Recent research has delved into the utilization of machine learning techniques for SLE analysis, customizing their methodologies to the specific dataset under investigation [22,43,44]. For example, Jorge et al. [20] utilized ML techniques to predict the hospitalization of SLE patients.

In the present study, the RF method, among all the ML classifiers employed, exhibited the most robust classification performance. It demonstrated superior accuracy levels and facilitated the identification of immunodeficiency patterns within the SLE population. This method offers scalability, rapid execution, and other beneficial features that enhance its classification capabilities [45]. ML models possess the capability to evaluate multiple variables and their interrelationships concurrently, accommodating non-linear patterns in the development of predictive systems [45]. Furthermore, we conducted a comparative analysis of our proposed system’s performance against various ML algorithms documented in Table 2, Table 3, Table 4 and Table 5. Notably, the RF method exhibited a substantial improvement, outperforming DT, BLDA and SVM, which demonstrated lower performance. Whilst the KNN method closely approached our proposed method, achieving AUC = 86% and Recall = 86%, RF demonstrated superior performance, surpassing both metrics with remarkable values of AUC and Recall, reaching around 94%. This notable improvement highlights the efficacy of the RF method in capturing complex patterns and enhancing the overall predictive capabilities.

Additionally, Figure 3 and Figure 4 illustrate a well-balanced performance graph for our proposed system, indicating minimal disparities between training and testing phases and no signs of overfitting. This establishes the system as a dependable tool, facilitating automated analysis to aid in the classification of SLE patients. Our results affirm the efficacy of the RF system in precisely predicting SLE patients, establishing it as a valuable tool for supporting SLE diagnosis.

## 5. Conclusions

In conclusion, due to the complexity of this elusive autoimmune disease, the use of ML algorithms such as RF is critical for the classification and rapid detection of patients with SLE flares. SLE presents with a range of challenging symptoms that are particularly difficult to diagnose accurately in its early stages. The intricate relationship between infections and autoimmunity in SLE underscores the critical need for preventative measures and the early detection of infections in SLE patients exhibiting heightened susceptibility. This integrated approach aims to address the multifaceted challenges of SLE, providing a more holistic understanding for improved patient care.

RF’s proficiency in handling diverse datasets and extracting intricate patterns makes it well-suited for identifying subtle indicators of SLE. The algorithm’s swift information processing enables quick detection, allowing for timely intervention and personalized treatment plans for SLE patients. Given the rarity and importance of SLE, the use of RF and similar ML approaches not only improves the diagnostic accuracy of SLE activity, but also contributes to improved patient outcomes, long-term monitoring, and a more effective healthcare management strategy for this devastating disease.

Thus, this investigation delves into the optimal ML technique for identifying patterns of immunodeficiency within the SLE population. It establishes that an ML system serves as a highly accurate tool for identifying diminished levels of immune parameters in individuals at a significantly elevated risk of experiencing both infections and, consequently, SLE flares. Moreover, the RF-based system proposed surpasses the performance of other studies, evident in a larger AUC, thereby affirming its superior predictive accuracy.

## Figures and Tables

**Table 1:** Characteristics of patients with immunodeficiency patterns.

Characteristics of Patients with Immunodeficiency Patterns	
N	77
Median age (years)	52
Female/Male	68/9
SLE evolution time (years)	14
Corticosteroids (n)	50 (64.9%)
Immunosuppressants (n)	25 (32.4%)
Hydroxychloroquine (n)	37 (48%)
Severe infections (n)	51
**Immunodeficiency patterns (n)**	
Leucocytes (<4000 cL/μL)	9
Lymphocytes (<1500 cL/μL)	28
Neutrophils (<1800 cL/μL)	9
CD3 (<700 cL/μL)	10
CD4 (<300 cL/μL)	6
CD8 (<200 cL/μL)	3
CD19 (<100 cL/μL)	23
NK (<90 cL/μL)	13
IgG (<870 mg/dL)	17
IgG1 (<383 mg/dL)	3
IgG2 (<242 mg/dL)	36
IgG3 (<22 mg/dL)	16
IgG4 (<4 mg/dL)	7
IgA (117 mg/dL)	8
IgM (<60 mg/dL)	20
C3 (<90 mg/dL)	13
C4 (<10 mg/dL)	6

**Table 2:** The table summarises the values of BA, recall, specificity, precision and AUC for variables IgG, IgG2, IgG3 and IgG4.

**IgG.**					
**Methods**	**BA**	**Recall**	**Specificity**	**Precision**	**AUC**
SVM	80.85	80.95	80.76	80.28	80.00
BLDA	78.11	78.20	78.02	77.55	78.00
DT	83.85	83.95	83.75	83.25	83.00
LR	70.02	69.75	68.84	68.95	68.42
RF	93.96	94.07	93.85	93.29	94.00
KNN	86.38	86.48	86.28	85.76	86.00
**IgG2.**					
**Methods**	**BA**	**Recall**	**Specificity**	**Precision**	**AUC**
SVM	81.85	81.95	81.76	81.27	81.00
BLDA	77.37	77.46	77.28	76.82	77.00
DT	83.16	83.26	83.06	82.57	83.00
LR	69.51	69.24	68.33	68.44	68.42
RF	94.58	94.69	94.47	93.90	94.00
KNN	85.99	86.09	85.89	85.38	86.00
**IgG3.**					
**Methods**	**BA**	**Recall**	**Specificity**	**Precision**	**AUC**
SVM	81.56	81.66	81.47	80.98	81.00
BLDA	79.16	79.25	79.06	78.59	79.00
DT	83.82	83.92	83.72	83.22	83.00
LR	69.44	69.17	68.27	68.38	68.42
RF	94.42	94.53	94.31	93.75	94.00
KNN	86.57	86.67	86.47	85.95	86.00
**IgG4.**					
**Methods**	**BA**	**Recall**	**Specificity**	**Precision**	**AUC**
SVM	81.35	81.45	81.26	80.77	81.00
BLDA	78.93	79.02	78.83	78.36	78.00
DT	83.26	83.36	83.16	82.67	83.00
LR	70.15	69.88	68.97	69.08	68.42
RF	94.50	94.61	94.39	93.83	94.00
KNN	86.07	86.17	85.97	85.46	86.00

**Table 3:** The table summarises the values of BA, recall, specificity, precision and AUC for variables IgM, NK, CD19 and CD3.

**IgM.**					
**Methods**	**BA**	**Recall**	**Specificity**	**Precision**	**AUC**
SVM	81.24	81.34	81.15	80.67	81.00
BLDA	78.11	78.20	78.02	77.55	78.00
DT	83.35	83.45	83.25	82.76	83.00
LR	69.86	69.59	68.68	68.79	68.42
RF	94.80	94.91	94.69	94.12	94.00
KNN	86.38	86.48	86.28	85.76	86.00
**NK.**					
**Methods**	**BA**	**Recall**	**Specificity**	**Precision**	**AUC**
SVM	81.06	81.16	80.97	80.49	81.00
BLDA	77.52	77.61	77.43	76.97	77.00
DT	84.84	84.94	84.74	84.24	84.00
LR	69.51	69.24	68.33	68.44	68.42
RF	94.75	94.86	94.64	94.07	94.00
KNN	86.51	86.61	86.41	85.89	86.00
**CD19.**					
**Methods**	**BA**	**Recall**	**Specificity**	**Precision**	**AUC**
SVM	82.21	82.31	82.12	81.63	82.00
BLDA	76.89	76.98	76.80	76.34	76.00
DT	84.04	84.14	83.94	83.44	84.00
LR	69.65	69.38	68.47	68.58	68.42
RF	94.34	94.45	94.23	93.67	94.00
KNN	85.24	85.34	85.14	84.63	85.00
**CD3.**					
**Methods**	**BA**	**Recall**	**Specificity**	**Precision**	**AUC**
SVM	81.46	81.56	81.37	80.88	81.00
BLDA	77.21	77.30	77.12	76.66	77.00
DT	84.16	84.26	84.06	83.56	84.00
LR	70.41	70.14	69.22	69.33	68.42
RF	95.12	95.23	95.01	94.44	95.00
KNN	86.38	86.48	86.28	85.76	86.00

**Table 4:** The table presents the F_1_ score, MCC, DYI and Kappa values for variables IgG, IgG2, IgG3 and IgG4.

**IgG.**				
**Methods**	**F_1_ score**	**MCC**	**DYI**	**Kappa**
SVM	80.61	71.74	80.85	71.98
BLDA	77.87	69.31	78.11	69.54
DT	83.60	74.40	83.85	74.65
LR	70.06	64.59	69.83	64.23
RF	93.68	83.37	93.96	83.65
KNN	86.12	76.65	86.38	76.90
**IgG2.**				
**Methods**	**F_1_ score**	**MCC**	**DYI**	**Kappa**
SVM	81.61	72.63	81.85	72.87
BLDA	77.14	68.65	77.37	68.88
DT	82.91	73.79	83.16	74.04
LR	69.54	64.12	69.32	63.76
RF	94.30	83.92	94.58	84.20
KNN	85.73	76.30	85.99	76.55
**IgG3.**				
**Methods**	**F_1_ score**	**MCC**	**DYI**	**Kappa**
SVM	81.32	72.37	81.56	72.61
BLDA	78.92	70.24	79.16	70.47
DT	83.57	74.38	83.82	74.62
LR	69.48	64.06	69.25	63.70
RF	94.14	83.78	94.42	84.06
KNN	86.31	76.81	86.57	77.07
**IgG4.**				
**Methods**	**F_1_ score**	**MCC**	**DYI**	**Kappa**
SVM	81.11	72.19	81.35	72.43
BLDA	78.69	70.03	78.93	70.27
DT	83.01	73.88	83.26	74.13
LR	70.19	64.72	69.96	64.35
RF	94.22	83.85	94.50	84.13
KNN	85.81	76.37	86.07	76.62

**Table 5:** The table presents the F_1_ score, MCC, DYI and Kappa values for variables IgM, NK, CD19 and CD3.

**IgM.**				
**Methods**	**F_1_ score**	**MCC**	**DYI**	**Kappa**
SVM	81.00	72.09	81.24	72.33
BLDA	77.87	69.31	78.11	69.54
DT	83.10	73.96	83.35	74.21
LR	69.90	64.45	69.67	64.08
RF	94.51	84.12	94.80	84.40
KNN	86.12	76.65	86.38	76.90
**NK.**				
**Methods**	**F_1_ score**	**MCC**	**DYI**	**Kappa**
SVM	80.82	71.93	81.06	72.17
BLDA	77.29	68.78	77.52	69.01
DT	84.59	75.28	84.84	75.53
LR	69.54	64.12	69.32	63.76
**Methods**	**F_1_ score**	**MCC**	**DYI**	**Kappa**
RF	94.46	84.07	94.75	84.35
KNN	86.25	76.76	86.51	77.02
**CD19.**				
**Methods**	**F_1_ score**	**MCC**	**DYI**	**Kappa**
SVM	81.97	72.95	82.21	73.19
BLDA	76.66	68.23	76.89	68.45
DT	83.79	74.57	84.04	74.82
LR	69.68	64.25	69.45	63.89
RF	94.06	83.71	94.34	83.99
KNN	84.98	75.63	85.24	75.89
**CD3.**				
**Methods**	**F_1_ score**	**MCC**	**DYI**	**Kappa**
SVM	81.22	72.28	81.46	72.52
BLDA	76.98	68.51	77.21	68.74
DT	83.91	74.68	84.16	74.93
LR	70.45	64.96	70.22	64.59
RF	94.83	84.40	95.12	84.68
KNN	86.12	76.65	86.38	76.90

## Data Availability

The datasets used and/or analyzed during the present study are available from the corresponding author on reasonable request.
